# Biosynthesis and Extraction of Chlorophyll, Carotenoids, Anthocyanins, and Betalaine In Vivo and In Vitro

**DOI:** 10.3390/cimb46090633

**Published:** 2024-09-23

**Authors:** Xinxin Yu, Hao Wang, Xingchun Xiang, Jingjing Fu, Xin Wang, Yuanhang Zhou, Wang Xing

**Affiliations:** 1 Key Laboratory of Sugar Beet Genetics and Breeding, College of Advanced Agriculture and Ecological Environment, Heilongjiang University, Harbin 150080, China; xinxinyuxx@163.com (X.Y.); wh2577002369@163.com (H.W.); xcxiang99@163.com (X.X.); bling199904@163.com (J.F.); wl190414@163.com (X.W.); 2 National Beet Medium-Term Gene Bank, Heilongjiang University, Harbin 150080, China; 3 Xinjiang Academy of Agricultural Sciences, Urumqi 830091, China; zhouyh1228@163.com

**Keywords:** biosynthesis, extraction, chlorophyll, carotenoids, anthocyanin, betalaine

## Abstract

As natural bioactive compounds, plant pigments play crucial roles not only in plant phenotype, growth, development, and adaptation to stress but also hold unique value in biotechnology, healthcare, and industrial applications. There is growing interest in the biosynthesis and acquisition of plant pigments. Thus, this paper explores emerging extraction methods of natural pigments and elucidates the biosynthesis pathways of four key plant pigments, chlorophylls, carotenoids, anthocyanins, and betalaine in vivo and in vitro. We comprehensively discuss the application of solvent, supercritical fluid [extraction], ultrasonic, and microwave-assisted extraction techniques, as well as introducing key enzymes, precursors, and synthetic pathways involved in pigment synthesis. δ-Aminolevulinic acid represents a pivotal initiating enzyme for chlorophyll synthesis, whereas isopentenylpyrophosphate, (IPP) and dimethylallyl pyrophosphate, (DMAPP) are closely associated with carotenoid biosynthesis. Phenylalanine and tyrosine are critical substances for anthocyanin and betalaine synthesis, respectively. Hence, crucial genes such as *chlI*, *crtB*, *PGT8*, *CYP76AD1*, and *BvDODA* can be employed for heterologous biosynthesis in vitro to meet the demand for increased plant pigment amount. As a pivotal determinant of plant coloration, an in-depth exploration into the high-quality acquisition of plant pigments can provide a basis for developing superior pigments and offer new insights into increasing pigment yield.

## 1. Introduction

Plant pigments usually refer to chlorophyll, carotenoids, anthocyanins, and betalaine [1]. Chlorophyll is the primary photosynthetic pigment of green plants, which is employed in the conversion of solar energy into chemical energy. Chlorophyll can be classified into five distinct types, including chlorophyll a (Chl a), and chlorophyll b (Chl b) [2,3]. Carotenoids are naturally synthesized by all photosynthetic organisms and non-photosynthetic organisms, such as eukaryotes, archaea, and bacteria, all of which exhibit complex carotenoid metabolic processes [4]. Carotenoids can transfer the captured light energy to chlorophyll a, which can assist in photosynthesis [5], and can participate in regulating plant growth and development; as natural antioxidants and biosynthetic precursors of vitamin A (retinol), carotenoids are involved in human nutrition and health, preventing the occurrence of diseases [6]. Flavonoids are important non-nitrogenous plant pigments [7]. Research indicates that despite the similar distribution and functions of anthocyanins and beet pigments in plants, there is limited evidence suggesting these compounds have ever coexisted in the same plant species [8,9,10]. Anthocyanins are a type of flavonoid, whereas betalaine, a nitrogen-containing pigment derived from tyrosine, includes betacyanin and betaxanthin [11]. Anthocyanins are responsible for the red, purple, or blue pigmentation in plants and fruits, with the intensity of this coloration depending on the position of substituents on the B ring [12,13].

Natural pigments contain a large number of vitamins, minerals, plant active compounds, and other nutrients, and exhibit various biological activities including antioxidant, anti-inflammatory, anti-cancer, neuroprotection, and cardiovascular protection [14,15]. Therefore, plant pigments have great potential in the cosmetics, pharmaceuticals, and food industries [15]. In recent years, driven by increasing health awareness and the discovery of new pharmacological effects associated with various natural pigments, natural colors are facing a rapidly growing global market [16]. However, traditional production methods are source-dependent and extraction is a key step in recovering purified bioactive pigments from specific plants. Conventionally, the extraction of natural plant pigments typically involves solid–liquid extraction, where plant tissues are processed by grinding and soaking to facilitate the diffusion of the pigments into the extraction solvent. The mechanical breakdown of tissues is associated with the release of cell walls and other cellular components, which require further purification. However, solvent extraction is limited by long processing times, low extraction rates, and high solvent requirements, and often constrained by the low content of target pigment compounds and their antagonistic effects on the environment and human health [17,18,19]. To address this issue, researchers have refined commonly used methods (e.g., Soxhlet extraction [20,21], extraction, etc.). New techniques, including ultrasound and microwave extraction, high-pressure extraction, supercritical fluid extraction, and electric field and pulsed electric field extraction, have also been introduced and are gradually being applied in this field [17,22,23].

Much effort has been made to increase pigment yields from “natural producers” by developing culture systems of plant cells and tissues in vitro, as well as optimizing microbial cultivation methods. Synthetic dyes demonstrate better and more stable performance in product development compared to natural dyes [15]. However, the synthetic pigment side effects and toxic effects [24] make people more focused on the safety of the product, so adopting more reliable development approaches is necessary. Synthetic biology makes it possible for the heterologous biosynthesis of pigments by designing and reconstructing novel biological modules and biosystems. This paper reviews recent advancements in major pigment extraction processes and discusses synthetic pathways for the four primary plant pigments. Finally, it addresses future challenges in the application safety of plant pigments.

## 2. Extraction of Plant Pigments

### 2.1. Liquid–Liquid Extraction or Solid–Liquid Extraction

The solvent extraction of plant pigments involves several steps such as drying, extraction, filtration, and evaporation. Depending on the nature of the material to be extracted, it can be classified as liquid–liquid extraction (LLE) or liquid–solid extraction (LSE) [25]. The commonly used methods of LSE include the impregnation method, infiltration method, decoction method, exudation method, Soxhlet extraction method, and steam distillation method. The extraction solvents typically used are water, ethanol, methanol, acetone, and other organic solvents. For decades, Soxhlet extraction has been described as a general chemical extraction technique [26]. Soxhlet extraction employs a special extraction apparatus that recycles the solvent in the biomass by condensing it in many cycles [25]. Whatever the extraction method, they share one common feature: the reliance on solvents. As a result, they all face a common drawback: the toxicity of organic solvents causes a certain amount of environmental damage [25,27,28]. For most plant pigments, the most commonly used solvents are water and aqueous alcohol. For beet glycosides, water is the optimal extraction solvent [15], and for anthocyanins, a combination of water and alcohol is the most suitable solvent [29]. In addition, other organic solvent systems can also efficiently extract plant pigments. Ashenafi [30] used different organic solvent systems (e.g., 80:20 *v*/*v* acetone:ddH_2_O, 80:20 *v*/*v* methanol:ddH_2_O water, 80:20 *v*/*v* acetonitrile:ddH_2_O water, and 40:40:20 *v*/*v* acetone:methanol:ddH_2_O) and found that β-carotene was only stable in the 80:20 (*v*/*v*) acetone:ddH_2_O solvent system after storage at standard refrigerator temperatures (4 to 8 °C).

### 2.2. Supercritical Fluid [Extraction], SFE

**Figure 1:**
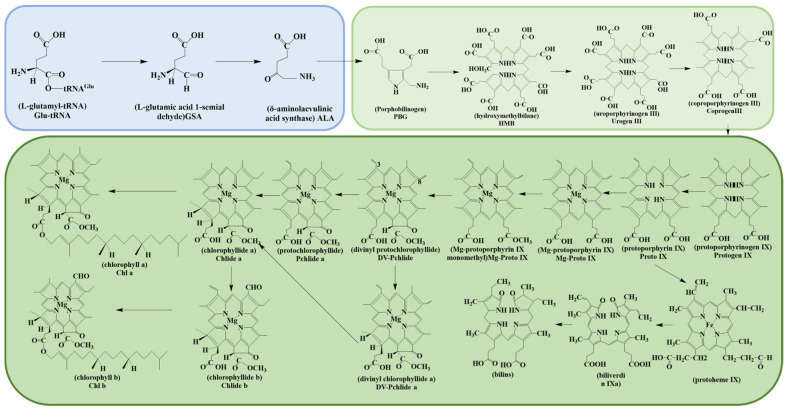
Chlorophyll biosynthesis pathway. In Figure 1, the color of chlorophyll synthesis was re-modified according to the content described. The light blue part represents the synthesis of ALA, and the light green part represents the synthesis of ALA to uroporphyrinogen III (Urogen III) and the final chlorophyll synthesis in the dark green box.

SFE technology relies on utilizing a substance called a supercritical fluid, which demonstrates characteristics of both gas and liquid states when kept above its critical temperature and pressure [31]. As shown in Figure 1, the advantage of this technology lies in its low cost and the ease with which supercritical fluids can be removed from the extracted compounds [29,32]. Among these, supercritical CO_2_ extraction is a green method that offers both economic and environmental benefits [33]. It has a low critical temperature (31.1 °C) and is non-toxic, making it suitable for extracting heat-resistant compounds. Research has shown that SFE is optimal with a CO_2_ content of 90%, 10% methanol (Me), 65 °C, 250 bar, 45 min, 9 mL/min. Under these conditions, the recovery rate of alizarin and its content in R. tinctorum extract (RE) were 1.34 g/kg and 6.42%, respectively [34]. Continuous extraction from *Butia capitata* fruits using supercritical CO_2_, ethanol and water gave a cumulative yield of 68.72% compared to 49.12% using the traditional methods. Overall, the extracts obtained using the SFE method demonstrated a higher antioxidant capacity for carotenoids, phenolic compounds, and unsaturated fatty acids compared to those obtained by conventional methods [35].

### 2.3. Correct to Ultrasound-Assisted Extraction, UAE

UAE is applicable for isolating target compounds from complex plant samples [23,36]. In Wang et al.’s study [37], utilizing ultrasonic extraction technology, natural yellow pigments were extracted from Physalis pubescens L. with the optimal parameters of 29.21% ultrasonic power, 14.41 min ultrasonic time, and 10.55 s ultrasonic interval, resulting in a yield of 0.193%. Furthermore, the use of corn oil as a green solvent for ultrasound-assisted extraction, in regard to antioxidants with color development results showed UAE > Microwave-Assisted Extraction, MAE > CE compared to MAE and conventional extraction, where UAE had higher TCC, DPPH, and TPC values [38]. Chesnokova et al. [39] reached the conclusion that modifying the ultrasonic-assisted time, temperature, power, and frequency of water and ethanol extraction of anthocyanin pigment resulted in a 22% increase in the yield of pigment in an aqueous solution. Surmanidze et al. [40] used sunflower seed oil as an alternative solvent to extract lycopene from Elaeagnus umbellata fruit by ultrasound-assisted extraction. Compared to the theoretical yield using a hexane–acetone–methanol (2:1:1) mixture as a reagent, the lycopene yield from Elaeagnus umbellata fruits can reach up to 85%. Carotenoids were extracted from the peel of the peach palm (*Bactris gasipaes* Kunth) fruit using soybean oil as the solvent. The extraction conditions were optimized to 48 °C for 28 min with a solid–solvent ratio of 0.0037. This process resulted in a carotenoid yield of 151.50 mg/100 g from the desiccated fruit peel [41]. Čulina et al. [42] optimized the UAE of 70% (*v*/*v*) ethanol, determining the following optimal conditions: ultrasonic power at 60%, temperature at 50 °C, and extraction time of 20 min. Carotenoids were extracted from *Cucumis melo* L. using a hexane–acetone (80:20) mixture at the optimal ratio, and the operating conditions were 10 min and 100% amplitude. The extraction rate was 124.61 ± 3.82 μg/g [43]. Ordoñez-Santos [41] et al. obtained 4.2 of betacyanin and 2.8 mg/g of betaxanthin by UAE at 52 °C and 37 °C, 90 min, 75% ethanol.

### 2.4. Microwave-Assisted Extraction, MAE

MAE can improve the quality and quantity of the extracted products due to the use of fewer solvents and shorter high-temperature exposure time [44,45]. The most effective technique for extracting fucoxanthin from cylindrical algae is MAE. The variables affecting MAE include microwave duration, power, temperature, and the type of algae [46]. Pretreated beetroots subjected to MAE using different microwave power levels (200, 400, and 600 W) and solvents (water, ethanol, and ethanol + citric acid) yielded betacyanin contents ranging from 23.77 to 59.28 mg/100 g [44]. Silva et al. [47] initially determined the optimal extraction conditions to be 30 min at 30 °C, with an ultrasonic power of 83 watts and a solid–liquid ratio of 75 mL/g. Compared with orbital shaking extraction (OSE), UAE demonstrated superior outcomes. Table 1 compares the advantages and disadvantages of Soxhlet extraction with the emerging techniques for extracting the three pigments. In conclusion, identifying the most suitable solvent and optimal extraction conditions is crucial. Optimizing the method ensures high extraction rates, functionality of the compounds, and adherence to green and sustainable chemistry principles [42].

## 3. Biosynthesis of Plant Pigments

### 3.1. Biosynthesis Pathways of Plant Pigments

#### 3.1.1. Chlorophyll

As shown in Table 2 and Figure 1, chlorophyll biosynthesis is a component of tetrapyrrole metabolism [56]. The process of chlorophyll synthesis can be divided into two distinct phases [57]. The initial stage involves the production of δ-aminolevulinic acid (ALA). Glutamic acid (GLU) is first catalyzed by Glu-tRNA Synthetase (GluRS), forming L-glutamyl-tRNA(Glu-tRNA). The intermediate is then reduced to form l-glutamic acid 1-semialdehyde (GSA) [58,59,60]. Lastly, GSA undergoes an isomerization, catalyzed by glutamate-1-semialdehyde aminotransferase (GSAT), to produce ALA [61]. ALA is a pivotal precursor in the biosynthesis of porphyrins, including chlorophyll and heme, in all organisms [62].

The second phase involves the transformation from protoporphyrin IX (proto IX) to chlorophyll. Tetrapyrroles, such as chlorophyll, heme, and siroheme, share a series of common steps from ALA to Urogen III in the downstream metabolic flux of ALA [60]. First, ALA molecules synthesize proto IX, which is then converted to magnesium-protoporphyrin IX catalyzed by magnesium chelatase (MgCH) [67]. Then, magnesium-protoporphyrin IX methyltransferase (MgPMT) catalyzed the generation of magnesium-protoporphyrin IX methyl ester through the methylation of magnesium-protoporphyrin IX. Following this, through the action of ring cyclase and two rounds of reduction and one round of oxidation reactions, chlorophyllide b (Chlide b) is generated. Finally, in the presence of chlorophyll synthase, chlorophyll a and b, along with the metabolite of protein geranylgeranyl pyrophosphate or phytol pyrophosphate, generate chlorophyll [68].

#### 3.1.2. Carotenoids

**Figure 2:**
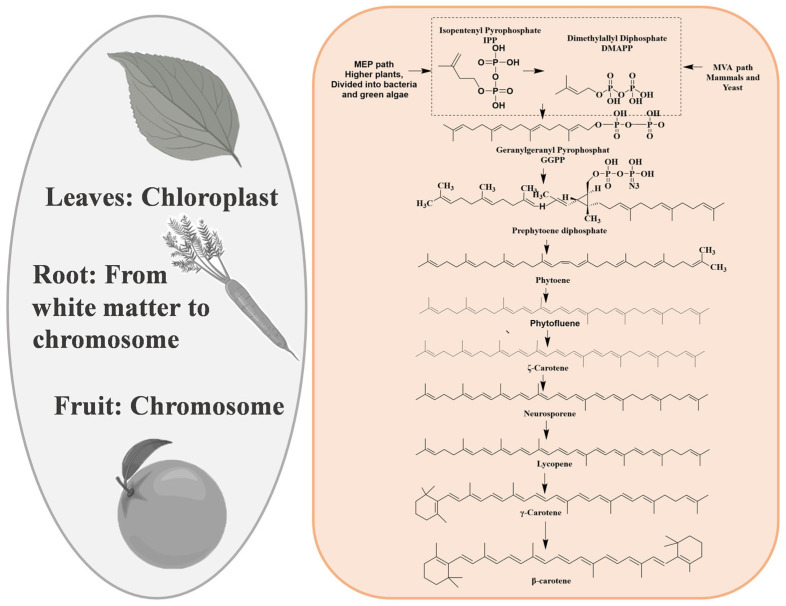
Carotenoid biosynthesis pathway. The pattern of carrots, oranges, and leaves in the figure comes from Figdraw. The imaginary part of the diagram the carotenoid C5 precursor IPP and DMAPP.

Carotenoids are a class of C40 lipophilic isoprene compounds composed of eight isoprene blocks, typically containing 40 carbons in their polyene skeleton [69,70,71,72]. As illustrated in Figure 2 and Table 1, in higher plants, carotenoids originate from C5 precursors IPP and DMAPP, which are predominantly derived from the MEP (plastidial methylerythritol 4-phosphate) pathway [73,74]. IPP and DMAPP, catalyzed by enzymes such as isopentenyl-diphosphate δ-isomerase (IDI), farnesyl diphosphate synthase (FDPS), and geranylgeranyl diphosphate synthase (GGPPS) [75] result in the production of the carotenoid precursor geranylgeranyl diphosphate (GGPP) [76]. Subsequently, GGPP is catalyzed by phytoene synthase (PSY) [77] to form the first synthesized colorless C40 carotenoid, phytoene [78,79]. Misawa et al. [80] first demonstrated that only one gene product (*CrtI*) is required to convert octahydrolycopene to lycopene. Lycopene is a major branch point in carotene biosynthesis and serves as a precursor for cyclic carotenoids such as γ-carotene, β-carotene, torularhodin, and astaxanthin [81]. Then, α-carotene is generated through the action of lycopene ε-cyclase (LCYE) and lycopene β-cyclase (LCYB). If only LCYB is involved, then only β-carotene is produced [82].

#### 3.1.3. Anthocyanins

**Figure 3:**
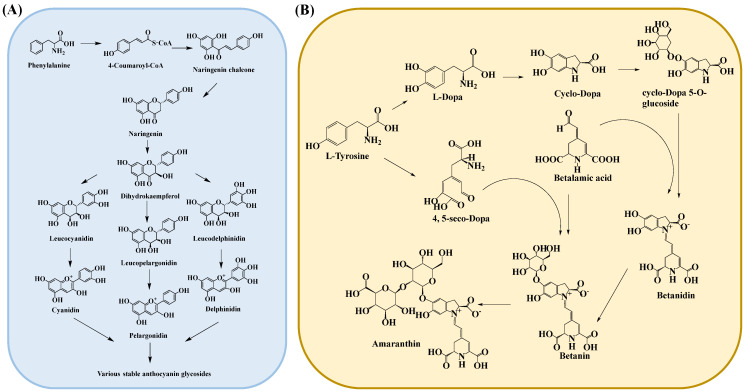
Biosynthesis pathways of anthocyanin (**A**) and betalaine (**B**).

During the biosynthesis of anthocyanins, phenylalanine serves as the precursor [83]. As shown in Figure 3A and Table 1, phenylalanine (Phe) is deaminated by phenylalanine ammonia-lyase (PAL), which is then oxidized to 4-coumaric acid by cinnamate 4-hydroxylase (C4H), and finally activated to 4-coumaroyl-CoA (4CL) by the addition of coenzyme A [84,85]. The activity of chalcone synthase (CHS) represents the initiation of a specific flavonoid pathway. The enzyme is responsible for the formation of chalcones by one molecule of 4-coumaroyl-CoA and three molecules of malonyl-CoA, which results in the formation of two phenyl rings (i.e., A and B rings) of the flavonoid skeleton (C6C3-C6). Chalcone isomerase (CHI) then catalyzes the formation of heterocyclic C, which produces naringenin (flavonoids) as an intermediate compound. Subsequently, naringenin is subjected to hydroxylation by F3H (flavanone 3β-hydroxylase), resulting in the formation of dihydroflavonol [86]. Finally, the colorless dihydroflavonol is converted into a variety of colored anthocyanins by dihydroflavonol-4-reductase (DFR) and anthocyanidin synthase (ANS). Flavonoid 3-O-glucosyltransferase (UFGT) catalyzes the glycosylation of the colored anthocyanins, resulting in the formation of stable, pigmented anthocyanin glycosides after glycosylation, methylation, or acylation modification [13,87].

#### 3.1.4. Betalaine

Beet pigments (Betalaine) can be categorized into betacyanins and betaxanthins [88]. As illustrated in Figure 3B and Table 1, betalaine is generated from tyrosine, which serves as the precursor in this process. Tyrosine undergoes hydroxylation by tyrosine hydroxylase (TOH) to form L-DOPA [89]. CYP76AD1 has been demonstrated to play a pivotal role in both the formation of cyclo-DOPA and the catalytic hydroxylation of tyrosine to L-DOPA [90]. Once L-DOPA is formed, it can be converted into betalamic acid (BA), a key intermediate responsible for the chromophore common to all betalaine [91]. Following the synthesis of L-DOPA, the biosynthesis of betalaine can be divided into two distinct branches. First, L-DOPA is catalyzed by DOPA-OX to generate dopaquinone, which is cyclized to form cyclo-DOPA. Cyclo-DOPA and betalaine then spontaneously form betacyanins. In the second branch, L-DOPA is catalyzed by 4,5-dioxygenase (DODA) into 4,5-seco-dopa [92], which then spontaneously condenses with amino acids or other amino groups to generate betalaine.

### 3.2. Heterologous Synthesis of Plant Pigments

#### 3.2.1. Chlorophyll Synthesis In Vitro

According to the known pathway of chlorophyll synthesis, Ipekoğlu et al. [93] cloned the chlorophyll a synthase gene *chlG* and successfully transformed it into *R. sphaeroides* to realize the expression of chlorophyll synthesis pathway in bacteria. Chen et al. [79] heterologously expressed genes involved in chlorophyll and carotenoid biosynthesis (*chlI*, *chlD*, *chlH*, *gun4*, *chlM*, *bciB*, *chlP*, *chlG*, *acsF*, *crtE*, *bchN*, *bchB*, *bchL*, *bchC*, *bchX*, *bchY*, *bchZ*, *bchF*, *bchG*, *crtI^Rs^*, *crtB^Rs^*, *dxs*, *crtY^Pa^*, *crtI^Pa^*, and *crtB^Pa^*) in *E. coli*, enabling *E. coli* to produce both chlorophyll and carotenoids simultaneously. Moreover, they modified the light-dependent chlorophyll a pathway in *E. coli* to function in the dark. For plants themselves, which naturally contain substantial amounts of chlorophyll, Liu et al. [94] observed that albino tea varieties employ seasonal greening as a survival strategy. They reported that CsRVE1 can directly bind to the promoters of CsLhcb, CsCHLH, and CsPORA, thereby increasing chlorophyll accumulation in tea leaves. The CsCHLI gene has been demonstrated to restore the lethal yellowing phenotype caused by the atchli1 mutation in different Arabidopsis thaliana lines, resulting in increased chlorophyll a and b [95]. In conclusion, mining and analyzing the key genes involved in chlorophyll synthesis across different plants could make significant contributions to enhancing the yield of synthetic chlorophyll in the future.

#### 3.2.2. Carotenoids Synthesis In Vitro

With the continuous progress of biotechnology, the methods for heterologous synthesis of carotenoids have become more and more sophisticated. *Blakeslea trispora* and *Phycomyces blakesleeanus* are natural carotene-producing fungi, and are also considered to be representative pathways for industrial-scale microbial production of carotenoids [96]. In the optimized culture of *B. trispora*, the laboratory-scale β-carotene production reached 78.0 mg/g DCW [97]. Hattan et al. [98] integrated CaCCS and CaZEP into S·tag and designed their interaction on the oligomerization scaffold of the S protein in *E. coli*, synthesizing capsicum yellow, capsanthin, and cucurbitacin A, which are specific carotenoids of red pepper fruits.

Lycopene cyclase has been identified as the rate-limiting enzyme in carotenoid biosynthesis due to its strong substrate inhibition of lycopene. Ma et al. [99] employed protein engineering for lycopene cyclase and established a flow regulator mediated by geranylgeranyl pyrophosphate synthase to increase carotenoid production. The Y27R mutant was capable of completely alleviating substrate inhibition without any reduction in enzyme activity. This led to a notable enhancement in β-carotene production with a selectivity of 98%. Using Schizochytrium sp., Zhang et al. [100] achieved a new record of 43-fold increase in β-carotene titer in a 5 L bioreactor (653.2 mg/L) by enhancing the precursor supply of geranylgeranyl diphosphate and regulating the carbon sources by limiting sugar fermentation. Sereti et al. [101] studied the fermentation behavior of two marine red yeasts (*R. paludigenum*) (2663 and 2664) cultured on glucose, fructose, sucrose, and glucose mixture (galactose), revealing the dynamic potential of the undeveloped yeast strain *R. paludigenum* 2663 as a robust producer of bioactive components. Fan et al. [102] introduced the NOG pathway to improve the supply of acetyl coenzyme A, and inhibited the ergosterol pathway to reduce the consumption of FPP. They introduced hSapB as a compensation tank for carotenoid storage, and designed the GAL expression system to function in YPD medium, ultimately obtaining the engineered strain JS-BE5-PEST, which produced 166.79 mg/L β-carotene and less than 0.5 mg/L lycopene in YPD medium.

#### 3.2.3. Anthocyanins Synthesis In Vitro

Postma et al. [103] assembled linear and circular chromosomes containing 41 genes in Saccharomyces cerevisiae. The resulting strain, IMF48, produced a titer of 0.049 ± 0.007 μM of pelargonidin-3-O-glucoside, establishing the first microbial cell factory for the de novo production of pelargonidin-3-O-glucoside (a type of anthocyanin). Ji et al. [104] screened a transcription factor, ChMYB1 (ChMYB90), which was significantly up-regulated during the fruit development of C. humilis. ChMYB1 was found to enhance the expression of *ChCHS* and *ChUFGT* genes by binding MBS (MYB binding element), resulting in a significant increase in the anthocyanin content in fruits. Furthermore, the exogenous application of abscisic acid (ABA) also promoted the expression of *ChMYB1* and the accumulation of anthocyanin.

#### 3.2.4. Betalaine Synthesis In Vitro

Wang et al. [105] assembled the pYB: CDD vector containing three betacyanin biosynthesis genes (*BvCYP76AD1S*, *BvDODA1S*, and *MjcDOPA5GTS*) and introduced it into carrot seedlings. The transgenic seedlings can produce betaine throughout the entire plant without substrate feeding. Metabolic engineering and fermentation optimization by Thomsen et al. [106] achieved the production of 1271 ± 141 mg/L beet glycosides and 55 ± 7 mg/L isobety glycosides by controlled fed-batch fermentation using glucose as a carbon source. Subsequently, Zhang et al. [107] used *S. cerevisiae* as a host to produce 28.7 mg/L betaine from glucose by screening and modifying the CYP76AD1-α branch protein, and optimizing the fermentation conditions, making the future metabolic engineering of betalaine possible. Betalaine can express color not only in plants themselves but also in other organisms such as yeast. By co-expressing three betalaine synthesis genes, cytochrome P450 enzyme CYP76AD1, DOPA 4,5-dioxygenase, and beet ligand 5-O-glucosyltransferase, it efficiently synthesized betalaine in silkworms. The betalaine in the cocoon layer can be extracted with water at room temperature, reaching a content of 14.4 μg/mg [108].

In total, chlorophyll, carotenoids, anthocyanins, and betalains each have distinct biosynthesis pathways reflecting their unique roles in plants. Chlorophyll biosynthesis begins with 5-aminolevulinic acid and involves complex steps including porphyrin formation and magnesium insertion. Carotenoids are synthesized from geranylgeranyl pyrophosphate through the isoprenoid pathway, with key steps involving lycopene formation and cyclization. Anthocyanins arise from the phenylpropanoid pathway, starting with phenylalanine and progressing through flavonoid intermediates to produce anthocyanidins. Betalains are synthesized from tyrosine via the betalamic acid pathway, involving the formation of betalamic acid and its coupling with cyclo-dopa. For in vitro synthesis, chlorophyll can be produced using chemical methods or through plant cell cultures, carotenoids via microbial fermentation or plant cell cultures, anthocyanins through enzymatic reactions or chemical synthesis, and betalains via plant cell cultures or microbial fermentation. These variations in synthesis methods reflect the diverse applications and production approaches for each pigment.

## 4. Improvement in Production of Plant Pigments

The extraction of natural pigments is further complicated by a number of external environmental factors, including light exposure, temperature changes, oxidation, pH, meso-electrode properties, the presence of metal ions, additives, and other variables. This intricate interaction of factors makes the extraction of natural pigments a difficult challenge [109]. Moreover, the direct extraction process often results in pigment loss and reduced purity. Therefore, more and more research prefers biosynthesized pigments as an alternative to plant-derived pigments. As shown in Table 3 the current methods for the biosynthesis of plant pigments. Involve microbial fermentation, microalgae culture, and plant tissue culture. Among these, plant cell culture and tissue culture allow for the direct extraction of pigments from plants.

## 5. Conclusions and Prospects

Plant pigments play a crucial role in a variety of cosmetic, and pharmaceutical products, especially in the food industry, where they are used as food additives. In recent years, many synthetic color additives have been banned due to their potential health risks resulting from excessive or improper usage. Therefore, enhancing the production of natural pigment and exploring new synthetic methods with improved safety profiles are particularly important. Despite the growing demand for pigments, there are still several challenges that need to be addressed: (1) the pigment extraction process is intricate and susceptible to factors such as pH, temperature, and time during the extraction process; (2) extracted plant pigments may have the taste of the plant itself, causing issues in the subsequent processing and utilization; (3) pigment yields are unstable, resulting in poor product quality and high costs; (4) the extraction process requires a large amount of biomass, and there will be waste generated, the management and reuse of these wastes have yet to be resolved.

The biosynthetic pathways for the four major plant pigments are now relatively well understood, particularly due to advances in omics technology. This has significantly enhanced the heterologous synthesis of pigments in vitro. According to their synthetic characteristics, the yield, purity, and stability of the pigments are also improved. Future developments in plant pigments should focus on the following aspects: deepening research into the factors influencing the synthesis of pigments, such as the pH, temperature, extraction agents, and culture media during cultivation and extraction processes; using transgenic or genome editing strategies to create new varieties with high pigment content; and establishing a co-culture system utilizing multiple engineered microbial strains to reconstruct the target biosynthesis. No matter what technology is used, future developments and applications of biosynthetic pigments should aim towards eco-friendly products that are safer, easily degradable, and free from adverse effects.

## Figures and Tables

**Table 1:** Comparison of control parameters, advantages, and disadvantages of four primary technologies.

Method	Control Parameter	Advantages	Limitations	Reference
Soxhlet Extraction	Eextraction solvent, extraction solvent reflux times (extraction time), ratio of solid to liquid, and extraction temperature	the instrument is low low-cost, simple, and can be used for multiple extractions;, the continuous flow of fresh solvents to the sample is beneficial to the extraction of the sample;, and the final extract does not need to be filtered	the process time is long, glassware is easy to damage, the open system is prone to solvent leakage, high boiling point temperature causes the thermal degradation of compounds, and cosolvent increases the cost	[28,48,49]
Supercritical Fluid [Extraction], SFE	Solvent, temperature, pressure, the addition of modifiers, solid to solvent ratio, and extraction time	high selectivity, it is suitable for highly sensitive applications, heat sensitive compounds are not easy to degrade, CO_2_ as a solvent is non-toxic and easy to obtain, and has a wide range of applications	the equipment is expensive, the operation is complex, CO_2_ is not suitable for some polar compounds, the parameter conditions are difficult, and the processing scale is small	[50,51,52]
Ultrasound-Assisted Extraction, UAE	Frequency, power intensity, amplitude, extraction time, temperature, type of ultrasonic, and treatment device (probe and bath)	shorten the extraction time, process automation, and reduce the consumption of organic solvents	requires professional equipment, only for laboratory scale (limited application), high energy consumption, extraction equipment conditions and ultrasonic conditions, and complicated operation procedures	[25,52,53]
Microwave-assisted Extraction, MAE)	Ssolvent feed ratio, solvent composition, moisture content, characteristics of plant samples, irradiation time, effect of stirring, microwave energy density, and extraction temperature	high efficiency and productivity, selective extraction of target compounds, high reaction rate, the product and process have high quality, reduce environmental pollution, and can reduce the degradation of heat-resistant components and reduce costs	the uneven heating leads to incomplete extraction, not suitable for reaction monitoring, high cost, complicated operation procedure, and limited application	[53,54,55]

**Table 2:** The four major pigments exist in plants and their representative structures.

Four Major Plant Pigments	Existence in Plants	The Most Representative Components	Reference
Chlorophyll	Higher plants and other organisms capable of photosynthesis	Chl a, Chl b as shown in Figure 1.	
Carotenoids	Yellow-orange vegetables and fruits, orange fruit, dark green vegetables, and lycopene	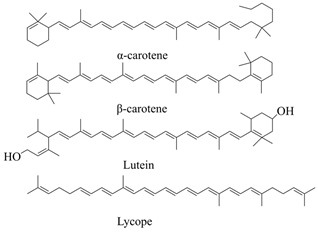	[63]
Anthocyanins	Tissues of purple sweet potato, grape, blood orange, red head cabbage, blueberry, eggplant, cherry, red berry, strawberry, mulberry, hawthorn, morning glory, Elderberries, etc.	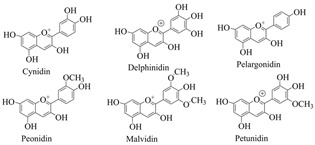	[64,65]
Betalaine	Chenopodiaceae, Amaranthaceae, Cactaceae, Mirabilis, and Phytolacca plants and some higher fungi.	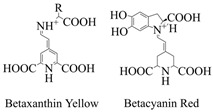	[66]

**Table 3:** Optimization in acquirement of natural pigments.

Methods of Improvement	Optimization Methods	Biological Excitation Factors	Reference
Plant cell culture and tissue culture	(1) Selection and development of high-yield cell lines; (2) optimizing cell culture process; (3) including the optimization of culture conditions; (4) recovering cells by immobilization; (5) redesign the bioreactor.	(1) Exogenous compounds secreted by microorganisms and insects when attacking plants; (2) exogenous compounds formed by plant enzymes degrading microbial cells, such as fungal and bacterial lysates and polysaccharides of microbial cell walls; (3) plant cell wall fragments degraded by pathogens; (4) intracellular proteins or compounds synthesized by plants in response to pathogen attack or abiotic stress (e.g., plant hormones); (5) non-biological elicitors (such as heavy metals, ultraviolet radiation, inorganic salts, etc.).	[16]
Microbial culture	(1) Medium; (2) process parameters; (3) extraction conditions.	(1) Cell growth; (2) nutritional factors (carbon source, nitrogen source, and C/N ratio); (3) microbial parameters (spore age, seed age, and inoculation age); (4) environmental conditions.	
Heterologous biosynthesis	(1) Engineering of rate-limiting biological components/factors (promoters, protein engineering to optimize enzyme properties, cofactors); (2) metabolic network engineering (supply of precursor substances to target products, enhancement of target product formation ability, inhibition of competitive pathways); (3) the engineering of cell system (external environment and cell expression).	Functional genes and responsible pathways.	
Co-culture system	Modularization	The biosynthetic pathway is divided into different host strains.	
